# Ruptured Tubo-Ovarian Pregnancy Presenting at 15 Weeks' Gestation

**DOI:** 10.1155/2022/5568711

**Published:** 2022-03-31

**Authors:** Daniel Keller, Matthew Morris, Ryan McLaughlin, David Evans, Michael Joyce

**Affiliations:** ^1^Department of Emergency Medicine, Virginia Commonwealth University Health System, Richmond, Virginia, USA; ^2^School of Medicine, Virginia Commonwealth University, Richmond, Virginia, USA

## Abstract

Ectopic pregnancies develop outside of the uterus and lead to significant maternal morbidity and mortality if they rupture. As the primary diagnostic tool for these presentations, ultrasound has a growing list of signs and measurements that help distinguish between intrauterine and ectopic pregnancies, the latter being exceedingly rare once a woman has entered her second trimester. The present case reports a series of Emergency Department visits by a woman carrying a second-trimester pregnancy—deemed intrauterine on transabdominal ultrasound due to gestational age and location—who developed massive hemoperitoneum and was diagnosed with a ruptured 15-week tubo-ovarian pregnancy on laparotomy. The discussion describes the sonographic findings that could have helped make the proper diagnosis, most notably mantle distance—the thickness of the myometrium surrounding the gestational sac—which would have correctly identified this pregnancy as ectopic.

## 1. Introduction

Ectopic pregnancy (EP) is a potentially life-threatening complication that occurs in 1-2% of pregnancies [1]. In the Emergency Department (ED), prevalence of EP tends to be significantly higher than population prevalence (8% and 2%, respectively) [2]. Ultrasound is the imaging modality of choice for early diagnosis of EP, a vital skill for emergency physicians due to the significant maternal morbidity and mortality associated with EP rupture and resulting hemorrhagic shock [1]. EPs can be suspected early when ultrasound of the expected intrauterine pregnancy shows an empty uterus. Most EPs implant in the fallopian tube and are particularly susceptible to rupture in the first trimester [3]. Tubal pregnancies surviving past 12 weeks are rarely documented [3]. The present case describes a series of ED visits by a woman carrying a second-trimester pregnancy—initially diagnosed as intrauterine on transabdominal ultrasound due to gestational age and location—who subsequently developed massive hemoperitoneum and was diagnosed with a ruptured 15-week tubo-ovarian pregnancy on laparotomy.

## 2. Case Report

**Figure 1:**
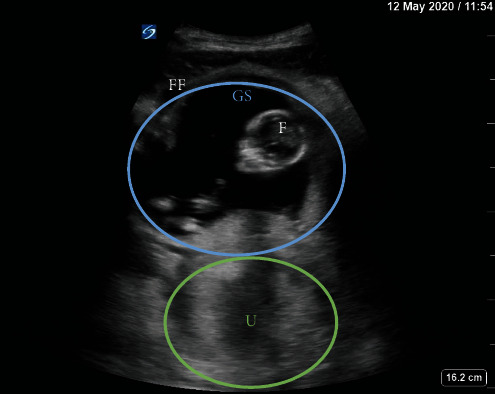
Bedside TAUS on visit 1. FF = free fluid; GS = gestational sac; F = fetus; U = uterus.

**Figure 2:**
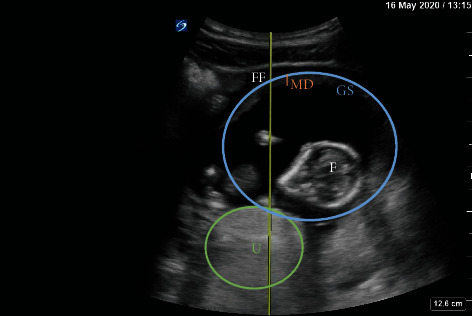
Bedside TAUS on visit 2. FF = free fluid; MD = mantle distance; GS = gestational sac; F = fetus; U = uterus.

**Figure 3:**
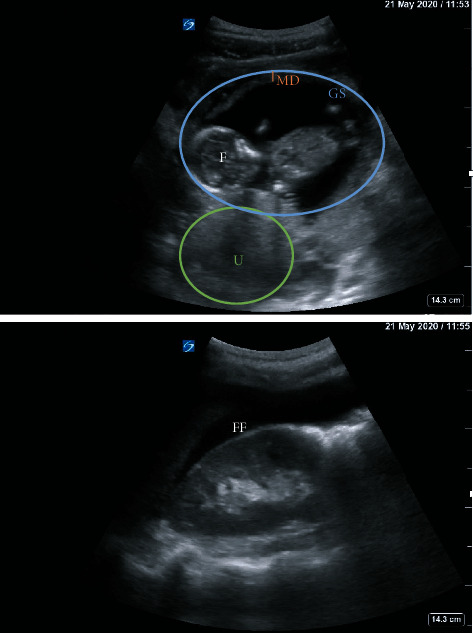
Bedside TAUS on visit 3. MD = mantle distance; GS = gestational sac; F = fetus; U = uterus; FF = free fluid around the liver tip.

**Figure 4:**
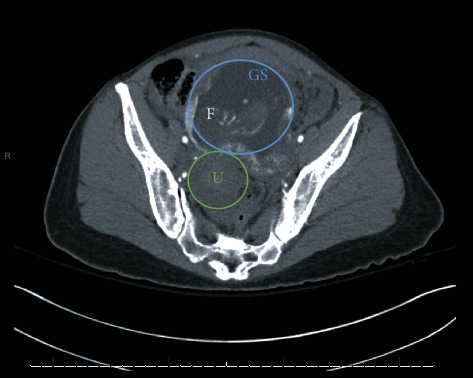
CTA showing fetal structures (F) within a gestational sac (GS) anterior to the uterus (U).

Over the course of 9 days, a 38-year-old G2P0020 female with history of asthma presented to the Emergency Department three times with abdominal pain. She reported her last menstrual period was two months ago and denied any vaginal bleeding. Her abdomen was tender on exam, but no peritoneal signs were appreciated. Her initial blood pressures (BP) were 96/61 mmHg on visit 1, 86/62 mmHg (visit 2), and 88/58 mmHg (visit 3). She was never tachycardic. Initial labs revealed a urinary tract infection and Trichomonas infection, for which she was prescribed antibiotics. During her first visit, bedside transabdominal ultrasound (TAUS) with the probe placed in the suprapubic region revealed a fetus estimated to be 14 weeks, 2 days old by biparietal diameter (Figure 1). The fetal heart rate (FHR) was 160 bpm and the pregnancy was determined to be intrauterine. Repeat ultrasounds on her second and third visits again showed an advanced pregnancy with appropriate fetal heart activity (Figures 2 and 3). Her BP and pain improved during her first two visits with fluids and symptomatic treatment. Each time, she reported feeling back to baseline, ambulated without difficulty, and was discharged with Obstetrics follow-up. On her third visit, free fluid was noted around the liver on ultrasound (Figure 3). Her hemoglobin on this visit was significantly lower: 8.5 g/dl on visit 1, 7.7 g/dl on visit 2, and 4.6 g/dl on visit 3. This precipitous drop in hemoglobin, combined with free intraperitoneal fluid on ultrasound, prompted further evaluation with computed tomography angiography (CTA) of the abdomen and pelvis. CTA showed fetal structures within a cystic cavity anterior to the normal uterus (Figure 4). Obstetrics was emergently consulted and the patient was taken for exploratory laparotomy. Surgery confirmed a 15-week tubo-ovarian pregnancy with significant associated hemoperitoneum. Right salpingo-oophorectomy was performed without complication. Following the operation, the patient was transferred to the Intensive Care Unit, and then discharged on hospital day 4.

## 3. Discussion

While there are multiple cases reporting ruptured first-trimester EPs, Liu et al. conducted a 2013 review and only identified 16 published cases of tubal pregnancies diagnosed in the second trimester, dating back to 1951 [3]. The authors explain that second-trimester EPs are exceedingly rare because of the vascularity and elasticity required to support such an advanced pregnancy. Given the risk of rupture and associated maternal morbidity and mortality, the use of ultrasound in the assessment of these EPs should leverage every possible advantage the modality offers. For instance, the patient in our present case had multiple clinical findings concerning for EP, but practitioners were repeatedly reassured by the age, location, and FHR noted on transabdominal ultrasound. Since characterizing pregnancies this way can still be misleading, there are a variety of other sonographic findings that can further indicate EP. A pseudo-gestational sac (collection of blood and debris in the endometrium), interstitial line sign (designates intramural implantation), and tubal ring sign (hyperechoic ring surrounding an extrauterine pregnancy) can all be suggestive of EP [4]. Tubal ring sign becomes a less specific finding for EPs in the second trimester [5], but one feature that will still maintain an accurate distinction between ectopic and intrauterine pregnancies is mantle distance, defined as the thickness of the myometrium surrounding the gestational sac, measuring mantle distance screens for asymmetric implantation suggestive of EP [6]. In a 2014 review of EP and ultrasound literature, Lewiss et al. concluded that a mantle distance <8 millimeters (mm) was a sensitive cut-off for emergency physicians to suspect EP [6]. Applied to this case, where myometrial thickness was repeatedly 5 mm, mantle distance would have provided a rapid measure designating this pregnancy as ectopic. We recommend that all emergency physicians who performed obstetric ultrasounds should include a measurement of the mantle distance.
